# The rumen microbial metaproteome as revealed by SDS-PAGE

**DOI:** 10.1186/s12866-016-0917-y

**Published:** 2017-01-07

**Authors:** Timothy J. Snelling, R. John Wallace

**Affiliations:** Rowett Institute, University of Aberdeen, Foresterhill, Aberdeen, AB16 5BD UK

**Keywords:** Cattle, Proteomics, Rumen, Sheep

## Abstract

**Background:**

Ruminal digestion is carried out by large numbers of bacteria, archaea, protozoa and fungi. Understanding the microbiota is important because ruminal fermentation dictates the efficiency of feed utilisation by the animal and is also responsible for major emissions of the greenhouse gas, methane. Recent metagenomic and metatranscriptomic studies have helped to elucidate many features of the composition and activity of the microbiota. The metaproteome provides complementary information to these other –omics technologies. The aim of this study was to explore the metaproteome of bovine and ovine ruminal digesta using 2D SDS-PAGE.

**Results:**

Digesta samples were taken via ruminal fistulae and by gastric intubation, or at slaughter, and stored in glycerol at −80 °C. A protein extraction protocol was developed to maximise yield and representativeness of the protein content. The proteome of ruminal digesta taken from dairy cows fed a high concentrate diet was dominated by a few very highly expressed proteins, which were identified by LC-MS/MS to be structural proteins, such as actin and α- and β-tubulins, derived from ciliate protozoa. Removal of protozoa from digesta before extraction of proteins revealed the prokaryotic metaproteome, which was dominated by enzymes involved in glycolysis, such as glyceraldehyde-3-phosphate dehydrogenase, phosphoenolpyruvate carboxykinase, phosphoglycerate kinase and triosephosphate isomerase. The enzymes were predominantly from the Firmicutes and Bacteroidetes phyla. Enzymes from methanogenic archaea were also abundant, consistent with the importance of methane formation in the rumen. Gels from samples from dairy cows fed a high proportion of grass silage were consistently obscured by co-staining of humic compounds. Samples from beef cattle and fattening lambs receiving a predominantly concentrate diet produced clearer gels, but the pattern of spots was inconsistent between samples, making comparisons difficult.

**Conclusion:**

This work demonstrated for the first time that 2D-PAGE reveals key structural proteins and enzymes in the rumen microbial community, despite its high complexity, and that taxonomic information can be deduced from the analysis. However, technical issues associated with feed material contamination, which affects the reproducibility of electrophoresis of different samples, limits its value.

**Electronic supplementary material:**

The online version of this article (doi:10.1186/s12866-016-0917-y) contains supplementary material, which is available to authorized users.

## Background

The rumen is the primary digestive organ in ruminants such as cattle, sheep, buffaloes and deer. It contains a vast number of anaerobic eukaryotic and prokaryotic microorganisms, which break down ingested feed materials to short chain fatty acids that are absorbed, to be used by the host animal for energy [1, 2]. The cellulolytic microbes are particularly important, because the host animal lacks the necessary enzymes to break down cellulose, which is abundant in forage diets [3]. The microbial cells formed during fermentation constitute the majority source of amino acids flowing to the gastric stomach [4]. Thus, ruminal fermentation is of vital importance to the nutrition of the animal. There are environmental problems associated with modern ruminant livestock production, principally the excretion of nitrogenous wastes and the emission of methane [5, 6]. Understanding the composition and activity of the rumen microbial community is therefore crucial if we are to improve productivity and to lessen the environmental impact associated with ruminant livestock. Furthermore, the rumen has a major influence on the health of the animal, so understanding the composition and function of the ruminal microbiota will also help to improve the health and welfare of the livestock [1, 7–9].

Traditional microbiological methods have largely given way to powerful non-cultivation methodologies, such as metagenomics and metatranscriptomics, in the study of complex microbial communities from environmental samples. Shotgun metagenomic sequencing has enabled a much deeper understanding of the composition of microbial communities and their gene contents, including those of the rumen [10–12]. Entire genomes of new, uncultured species have been assembled [10]. Novel enzymes have been extracted by so-called gene mining strategies [10, 13–15]. Most important for the livestock industry, we are beginning to understand the relations between microbial species and gene abundances and production characteristics, including methane emissions [16–18]. Metatranscriptomics describes the transcription of the genes to mRNA, which gives an impression of the activity of the microbial community rather than just its genetic complement [19]. It might be argued, however, that it is the combined output of transcription and translation, the metaproteome, that might tell us most about actual metabolic activity in the ecosystem.

Metaproteomic analysis aims to characterise the entire protein content of an environmental sample at a given point in time [20] Two main technical approaches are available in proteomics. The first is the well established 2D SDS-PAGE technology originated by O’Farrell [21]. Individual proteins are separated by isoelectric point in the first dimension and size in the second. The proteome is then visible when the gel is stained. Individual spots can be identified by peptide analysis following trypsinisation. Protein identification depends heavily on databases that generally do not include ruminal species. Activated sludge systems [20], anaerobic waste water [22], soil and sediments [23, 24], rhizosphere [25] and human faecal samples [26] have already been analysed by this method, but not until now the rumen. The second method utilises state-of-the-art mass spectrometric analysis of peptides derived by partial hydrolysis of protein mixtures without protein separation, so-called shotgun peptide sequencing. Many believe that the shotgun method, with the much larger volume of data generated, will supplant the gel-based method. Once again, the method relies upon peptide databases in which the great majority of ruminal species are not represented. The first analysis of the ruminal metaproteome using the latter methodology was published in 2015 [27].

The rumen shares some characteristics of the communities that have previously characterised by metaproteomics, in terms of microbial diversity and relative abundance of microorganisms and food materials in others, but it provides a unique challenge in the combination of these properties. The metaproteome will provide an alternative insight into the function of the rumen microbial community compared to the nucleic acid meta-omes, arguably one that might prove more useful as part of the campaign to lower methane emissions and to better understand the role of key enzymes involved in feed utilisation efficiency in ruminants. The aims of this study were to investigate the effectiveness of SDS PAGE methods for generating metaproteomic information from ruminal digesta and to evaluate the information that can be obtained by this method.

## Methods

### Animals and digesta sampling methods

Samples of ruminal digesta were obtained from dairy cows in Sweden and Finland, reindeer in Finland, and beef cattle and lambs in Scotland. Sampling was from live animals in the cases of dairy cows, reindeer and beef cattle and *post mortem* in the case of lambs. The dairy cows were kept according to licences granted by national regulatory authorities and the experimental protocols were scrutinised by local welfare committees.

Red dairy cows from Sweden received a diet containing a mixture of grass silage (632 g/kg DM) and barley (218 g/kg DM), with rapeseed expeller added as a protein supplement (100 g/kg DM). The diets were fed ad libitum as a total mixed ration.

Cows and reindeer from Finland were fed the same total mixed ration based on grass silage and concentrates (60:40 forage:concentrate ratio on a DM basis) at a restricted level of intake to meet maintenance energy and protein requirements.

Beef cattle kept at Easter Bush Farm, Midlothian, Scotland received 60% forage with 40% concentrate diet in which the main ingredient was barley (20%).

Lambs received a diet comprising 70% forage and 30% complete feed concentrate containing 22.6% barley, 4% wheat, 2% soya, minerals and supplements.

Ruminal digesta samples (4 ml) were taken manually from cows and reindeer in Finland and Sweden via a ruminal cannula, diluted in 8 ml PBS buffer containing 20% glycerol and transported to the laboratory on dry ice where they were stored at −80 °C. Fresh digesta was obtained from the beef cattle and lambs immediately after slaughter and stored in an insulated container prior to protein extraction.

### Sample processing

The digesta obtained from the lambs and cannulated reindeer and cows contained large amounts of dietary fibre. After thawing the sample, the coarse fibres were separated by gentle centrifugation (200 × *g*) and the supernatant retained. The remaining fibres were washed in a sodium phosphate and detergent buffer (50 mM pH 6.5, 0.1% (*v*/*v*) Tween 80) and the centrifugation process was repeated three more times. The pooled supernatant was centrifuged at 12,000 × *g* at 4 °C for 20 min to collect the enriched microbial fraction [28]. The dairy cow and beef cattle rumen fluid samples taken by gastric tube contained very little coarse fibre and were processed as received. After thawing, the samples were left to settle for 15 min to reduce any residual course fibre and feed particles. This step also reduced the number of protozoa, which would otherwise dominate the microbial proteome. The remaining fraction was aspirated and immediately centrifuged at 12,000 × *g* at 4 °C for 20 min. In all cases, the pellet was then resuspended in 1.5 ml lysis buffer based on Rabilloud [28] containing 7 M urea, 2 M thiourea, 4% CHAPS, 1% dithiothreitol and a protease inhibitor cocktail (Sigma Aldrich). Protein extraction was carried out using six rounds of 30 s bead beating with 3 min cooling on ice based on the DNA extraction protocol by Yu and Morrison [29].

Proteins were precipitated by adding 6 M trichloroacetic acid/80 mM DTT solution at a ratio of 1:3 protein extract, vortexing and incubating overnight at 4 °C. The tubes were centrifuged at 16,000 × *g* for 20 min at 4 °C and the supernatants were discarded. The pellets were washed twice in a −20 °C solution of 20% DMSO in acetone and twice in −20 °C acetone based on the protocol by Song et al. [30]. The pellet was resuspended in the Rabilloud buffer described previously and the concentration of total protein extract was assayed using the RC/DC method (Bio-Rad) against a BSA standard.

Visual inspection of the dissolved protein extract revealed some brown discolouration of the solution indicating the presence of dissolved organic compounds. This was most notable in the samples from the silage/concentrate fed reindeer and cow samples. Where this occurred, additional extraction methods to remove these organic compounds were attempted including; wash dilution, column filtration and phenol extraction. After centrifugation, the enriched microbial pellet was resuspended in a sodium phosphate wash buffer (50 mM pH 6.5) containing 0.1% *v*/*v* Tween 80. The solution was shaken gently for 5 min and the microbial pellet retrieved by centrifugation at 12,000 × *g*. This process was repeated three to four times, or until the supernatant was clear. Column filtration was carried out using an Amicon® Ultra-4 centrifugal filter (Millipore) 10 kDa molecular weight cut-off according to the manufacturer’s protocol. Phenol extraction was carried out subsequent to the TCA and acetone precipitation stage and based on the protocol by Wu et al. [25].

### Metaproteome analysis

Protein separation was carried out on individual samples using 1D and 2D SDS-PAGE using precast gels according to the manufacturer’s protocol (Bio-Rad). 100–300 μg protein in 325 μl Rabilloud buffer were applied to immobilised pH gradient (IPG) strips pH from 4–7 as standard after previous work with pH 3–10 IPG strips determined that the isoelectric points of most proteins were contained within this range. Gels were stained using Coomassie Blue. Visibly abundant protein spots were selected on the 2D gels for protein identification by LC-MS/MS. These were cut manually and the gel plugs destained and digested with trypsin. The resulting peptide solution was processed using an Ultimate nano LC system, with Famos autosampler and Switchos microcolumn (Thermo Scientific). Ionisation and mass spectra measurement was carried out using an AB Sciex Q-Trap triple quadrupole mass spectrometer. Total ion current data was submitted to the MASCOT server (Matrix Science) to identify the most likely protein hit from the NCBI nr database. The search criteria were: allowance of 0 or 1 missed cleavages; tolerance of ± 1.5 Da; fragment mass tolerance of ± 1.5 Da, trypsin as digestion enzyme; carbamidomethyl fixed modification of cysteine; methionine oxidation as a variable modification; and charged state as 2+ and 3+. Potential function was inferred from the annotation of the protein best hit provided by the NCBI nr database entry. This was carried out using MASCOT (Matrix Science Inc.), which uses a probabilistic scoring system for protein identification/inference adapted from the MOlecular Weight SEarch (MOWSE) algorithm.

## Results and discussion

### Effects of different sample types on electrophoresis

The ruminal metaproteome from a variety of species and sample types was characterised by SDS-PAGE using digesta samples obtained from a variety of ruminant species, including cattle, sheep and reindeer, fed different diets. The digesta samples were taken via cannula from the silage/concentrate fed cows and reindeer and the fresh samples taken from the forage/concentrate fed lambs contained a large proportion of coarse dietary fibre. For these samples, a method was adapted to remove the fibre and enrich the bacteria fraction using differential centrifugation and a wash dilution procedure [31]. In comparison, the samples from the forage/barley concentrate fed dairy and beef cattle taken by gastric tube contained very little in the way of plant fibre and were processed as received. The difference in sample quality was accepted to be a result of the methods and was not considered as a factor associated either with the individual animal or species. Different sampling methods - stomach tube, rumen cannula and slaughter – were used in this study, but no two methods were compared directly, therefore it is not possible to assess if sampling method had any influence on gel quality.

**Fig. 1:**
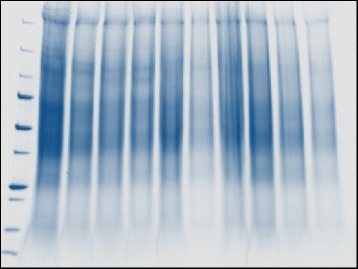
1D SDS-PAGE of ruminal digesta protein extract. Standard ranges 15–250 kDa. Lanes from left to right: Five reindeer and five cows from Finland (MTT)

**Fig. 2:**
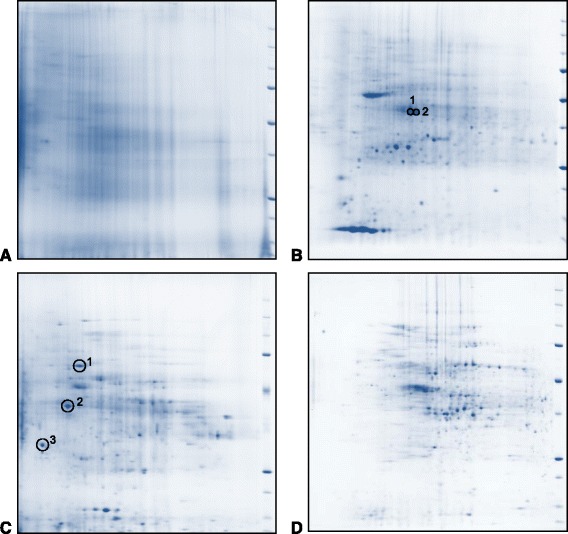
2D SDS-PAGE of proteins extracted from ruminal digesta. Digesta samples were obtained from different host species using different sampling methods, mixed with PBS/glycerol buffer and stored at −80 °C. In all gels size standards range from 10–250 kDa from bottom to top, isoelectric points (pI) range from pH 4–7 from left to right. **a**. Reindeer from Finland (MTT) fed silage forage based diet. Samples were obtained manually via ruminal cannulae. Protein extraction was carried out after bacterial enrichment by differential centrifugation and wash dilution stages. The gel shows severe protein degradation and spots are obscured by co-staining of humic compounds. **b**. Dairy cows from Sweden (SLU) fed a high protein diet. Samples were taken by intubation via ruminal cannula. Protein was extracted with no sample pre-processing. Spots identified: 1. Actin, *Entodinium caudatum*, GI: 3377675. Based on eight peptide matches, 36% coverage, theoretical size 41.7 kDa. 2. Actin, *E. caudatum*, GI: 3386579. Based on eight peptide matches, 34% coverage, theoretical size 41.7 kDa. **c**. Beef cattle from Scotland on a fattening high concentrate diet. Samples were taken by nasogastric intubation. Large particles were separated and removed by settling for 5 min before continuing to the protein extraction stages. Spots identified: 1. Methyl-CoM reductase McrA, *Methanobrevibacter smithii*, GI: 518094697. Based on five peptide matches, 12% coverage, theoretical size 61.1 kDa. 2. Methyl-CoM reductase beta subunit McrB, *M. ruminantium* M1, GI:288561184. Based on three peptide matches, 8% coverage, theoretical size 47.2 kDa. 3. 5,10-methylenetetrahydromethanopterin reductase, *M. ruminantium* M1 GI:288559826. Based on five peptide matches, 22% coverage, theoretical size 33.1 kDa. Additional proteins identified are described in Tables 1 and 2. **d**. *Post-mortem* digesta from lambs from Scotland fed on a finishing concentrate diet. Protein extraction was carried out on fresh samples after bacterial enrichment by differential centrifugation and wash dilution stages. Proteins identified are described in Tables 1 and 2

Samples were firstly subjected to 1D SDS-PAGE in order to anticipate possible problems associated with impurities in the samples (Fig. 1). The lack of clear banding in 1D SDS-PAGE was due partly by the complexity of the metaproteome and partly by the presence of contaminants, possibly humic compounds, co-precipitating with the proteins. This assumption was based on similar studies reporting co-staining with Coomassie blue of these compounds on SDS-PAGE derived from similar environmental samples [32]. Therefore, no attempt was made to identify proteins using LC-MS/MS from these gels. The lack of resolution of proteins was also evident in the 2D SDS-PAGE results of the dairy cow and reindeer digesta samples (Fig. 2). Here, contaminants co-stained by the Coomassie Blue consistently obscured proteins in all of the gels prepared from the protein extracts [33]. A typical feature of the 2D gel was a dark streak in the acidic region of the isoelectric focussing [24]. Even where it was possible to identify faint spots on these gels, there was not sufficient protein to identify using LC-MS/MS.

Repeated 2D SDS-PAGE gels from different extracts from the same sample produced highly reproducible results (see Additional file 1). In contrast, spot patterns were poorly reproduced between 2D gels of different samples, preventing any meaningful comparison of protein abundance between gels. Scans of the gels were loaded onto PDQuest analysis software (Bio-Rad) to align the gels and detect spots in an attempt to assess relative values of protein abundance. Setting the levels for fainter spots was hampered by high background staining and the complexity of the pattern. In most cases the 2D gels were dominated by a few highly abundant proteins with fainter spots at the limit of detection by the software. The estimation of protein loading on the gel was also affected by contaminants affecting the result of the absorbance readings taken as part of the RC/DC assay used to measure protein concentration (BioRad) [22].

The additional methods, including the wash dilution, column filtration and phenolic extraction steps carried out to eliminate or reduce humic compound contamination were all unsuccessful, and did not produce any clearly resolved 2D SDS-PAGE results. The use of DMSO during acetone precipitation was previously reported to reduce contamination in plant root proteins [30] and although the effect was inconclusive in the case of rumen digesta, it was used in all the extractions performed here.

### Identification of proteins on 2-D gels

**Fig. 3:**
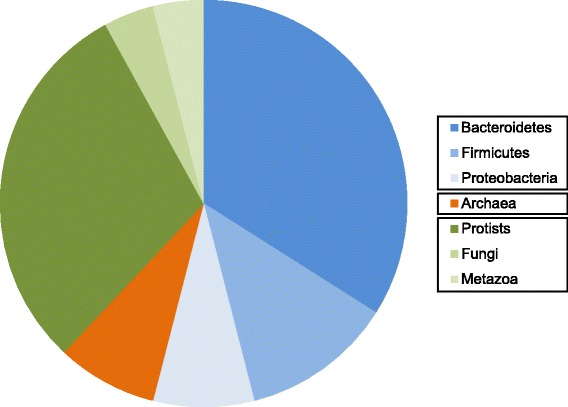
Taxonomy summary of rumen proteins. The taxon pie chart is based on the 50 unique proteins identified from the 2D gels shown in Fig. 2b, c and d and listed in Table 1. The chart shows the relative richness of unique proteins from bacteria (blue), archaea (orange) and eukaryotic (green) groups

Coomassie Blue was used to stain gels in all cases following the principle that any resolved protein bands or spots should yield sufficient material (10–100 ng) to identify using mass spectroscopy [34]. MASCOT MS/MS ion search results of the selected spots from the 2D SDS-PAGE gels are shown in Fig. 2b-d. Proteins were selected on the basis of highest MASCOT score and percent coverage. A number of MASCOT hits that gave human keratin or trypsin as a result were disregarded and considered as either contaminants for the former or the enzyme used for protein digestion for the latter. A taxonomic summary of the unique proteins found is shown in Fig. 3 and a complete list of the unique highly abundant proteins (by GI sequence identification number) from the successfully resolved gels is given in Table 1 (prokaryotic proteins) and 2 (eukaryotic proteins). The number of peptides mapping to the protein hit and the percent coverage gave a degree of confidence to the protein identity. Proteins identified from a single peptide and coverage less than 5% were excluded from the results. The theoretical (MW_t_) size was also compared to the position of the spot on the gel (MW_e_). However, in some cases the MW_t_ and MW_e_ value differed due to a partial protein reference sequence or the protein separating into subunits on the gel.

**Table 1:**
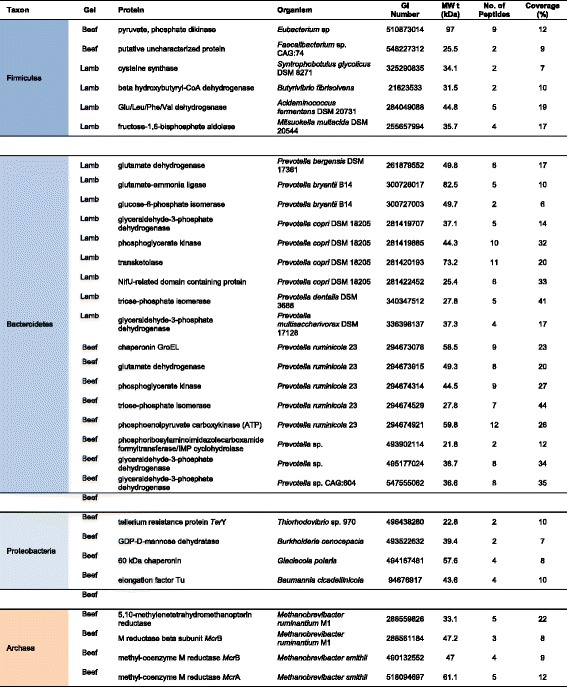
Abundant prokaryotic proteins from ruminal digesta from different species visualised by 2D SDS-PAGE and identified using LC-MS/MS

#### Protozoal proteins

The 2D SDS-PAGE of the rumen fluid taken from high concentrate fed dairy cows was dominated by a few very abundant proteins (Fig. 2b). LC-MS/MS identified these as actin, alpha and beta tubulin and axonemal isoforms of dynein light chains. All these proteins can be found in the cilia of rumen protozoa and taxonomic identification confirmed the rumen ciliate species *Entodinium caudatum* as the most likely source. While this group of microorganisms is not as abundant as the bacteria, they can make up a large proportion of the microbial biomass [1].

To date, there are no completed genome sequences for rumen ciliate species; consequently a reference proteome was not available. However, some 52 coding cDNA sequences obtained from functional screening and structural protein analysis of the rumen ciliate genus *Entodinium* were contained in the NCBInr protein database [35]. Five of the 15 eukaryotic structural proteins mapped to this small reference dataset with four others mapped to related ciliate genera: *Euplotes*, *Ichthyophthirius*, *Spathidium*, *Epiphyllum* and *Amphileptus* (Table 2). In the NCBI nr database used in the present study, reference sequences for similar structural proteins such as actin are abundant, originating from a wide range of organisms. The narrow range of taxa that the proteins mapped to here was an indication of the specificity of the amino acid sequence identity to rumen species.

**Table 2:**
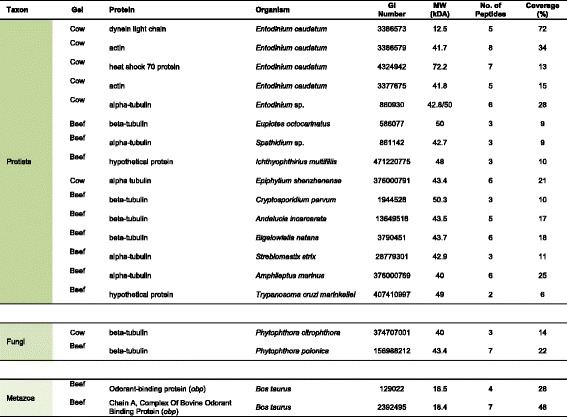
Abundant eukaryotic proteins from ruminal digesta from different species visualised by 2D SDS-PAGE and identified using LC-MS/MS

##### Bacterial proteins

To increase the abundance of the prokaryotic microbial proteins relative to the ciliate structural proteins, it was necessary to fractionate the samples by settling for 15 min. SDS-PAGE results with this step included are shown in Fig. 2c. This gel lacked the dominant spot pattern apparent in Fig. 2b and highly abundant proteins identified by LC-MS/MS from a broad range of organisms. Based on the best hits results provided by the MASCOT search of total ion current data, the majority of proteins originated from bacteria with others from the host, plants, fungi, archaea and protozoa.

Similar results were seen in the *post-mortem* samples from the lambs on the high concentrate diet (Fig. 2d). The dominant spot pattern indicating abundant ciliate structural proteins identified in gel 2B was not seen on the gel and no ciliate proteins were detected by LC-MS/MS. In this sample, the bacteria enrichment steps for these samples to remove larger contaminating particles used repeated dilution and centrifugation [31] and may well have resulted in the complete removal of ciliates.

Many of the prokaryotic proteins were central metabolic enzymes such as glyceraldehyde-3-phosphate dehydrogenase, phosphoenolpyruvate carboxykinase, phosphoglycerate kinase and triosephosphate isomerase, involved in central carbohydrate metabolism pathways and present in almost any cellular organism. However, in a similar manner to the ciliate structural proteins, the amino acid sequence identity of these enzymes was associated with of rumen prokaryote species that had been characterised previously in genomic studies [36] (Table 1). Bacterial phyla included Bacteroidetes, Firmicutes and Proteobacteria. The Firmicutes proteins were from a diverse range of species, some associated directly with the rumen or the human gut and some with anaerobic sewage environments, the latter being a result of the paucity of protein sequences of rumen microorganisms in the reference database. All the Bacteroidetes proteins were from species of *Prevotella* and accounted for over half of the total prokaryotic proteins. The dominance of *Prevotella* proteins reflected the abundance of this genus in the rumen [37, 38].

#### Archaeal proteins

The ruminal archaea are much lower in abundance than bacteria, on average approximately 5% of the bacterial population based on relative abundance of 16S rRNA subunit [17, 39, 40]. Two archaeal proteins were discovered here as dominant spots. 5,10-methylenetetrahydromethanopterin reductase (*mer*) from *Methanobrevibacter ruminantium* and methyl coenzyme M reductase beta subunit (*mcrB*) from *M. ruminantium* and *Methanobrevibacter smithii* are both important components of the pathway converting CO_2_ and H_2_ into methane [19]. The detection of these enzymes from a relatively small proportion of the microbial community highlights the importance of methane metabolism in the rumen. Ruminants produce abundant quantities of methane, up to 500 L/d in a dairy cow [6]. Methane is a greenhouse gas (GHG) with a global warming potential 28-fold that of carbon dioxide [41]. Methane production from ruminants accounts for the majority of the 37% of total GHG from agriculture in the UK [42]. Ruminal methanogenesis derives from fermentation by bacteria, protozoa and fungi, which produce short-chain fatty acids and H_2_; the latter which, with CO_2_, is the main substrate for methane formation by methanogenic archaea [6]. Understanding this complex process is vital if we are to develop methods to lower methane emissions from ruminants and thereby lessen the environmental impact of livestock agriculture. The present work indicates that metaproteomics may be a useful tool in achieving that aim.

### Perspective

Deusch and Seifert [27] made the first description of the ruminal metaproteome by shotgun peptide sequencing. 2-D gels clearly provide a visual image that the shotgun method does not. However, the power of the shotgun technique compares very favourably compared to 2D SDS-PAGE, in the sense that thousands of peptides were analysed in a non-selective way, whereas only a few hundred were analysed by SDS-PAGE here. Furthermore, the electrophoresis itself is subject to major problems that affect the comparison of different samples. Yet the ability to identify visible proteins on a gel has some merit, we believe. Both methods enable an analysis of the phylogenetic origin of a peptide/protein. It is extremely important to point out that the weakness of the databases with regard to the poor representation of true ruminal organisms is a handicap to both methods. As far as we are aware, there is no corresponding proteomic initiative to match the Hungate 1000 genomic project [43], which will enable precise assignment of gene sequences to phylogenetic taxa.

## Conclusions

Despite the taxonomic diversity of the rumen, a relatively small number of protein spots dominated the metaproteome. Co-precipitation of grass-derived contaminants had a critical effect on the outcome of the SDS-PAGE protein separation and visualisation, such that within-sample replication was excellent and between-sample replication was poor, lowering the value of SDS-PAGE as a tool to predict rumen function. Although the volume of data retrievable from 2D-SDS-PAGE was a couple of orders of magnitude less than shotgun peptide sequencing analysis [27], the conclusions on protein complement were qualitatively similar. 2D-SDS-PAGE, when successful, has the advantage of creating an image that can be compared with others visually. Enzymes from methanogenic archaea were among the most readily identifiable proteins, indicating a possible role for metaproteomics in exploring low-emitting phenotypes of ruminants, which in turn may enable mitigation of greenhouse gas emissions from farm livestock production by selective breeding.

## Additional file

Additional file 1: Comparison of four gels run using the same sample from a Swedish red cow. (DOCX 404 kb)

## Abbreviations

1D- and 2D-SDS-PAGE: One- and two-dimensional gel electrophoresis
DM: Dry matter
DMSO: Dimethyl sulfoxide
DTT: Dithiothreitol
GHG: Greenhouse gas
LC-MS/MS: Liquid chromatography followed by tandem mass spectrometry
PBS: Phosphate-buffered saline
