# Supplementary material for: The rumen microbial metaproteome as revealed by SDS-PAGE
Source: BMC Microbiol. 2017 Jan 7;17:9. doi: 10.1186/s12866-016-0917-y (PMC5219685; doi:10.1186/s12866-016-0917-y)

The rumen microbial metaproteome as revealed by SDS-PAGE

T J Snelling and R J Wallace

Additional file 1

Comparison of four gels run using the same sample from a Swedish red cow.


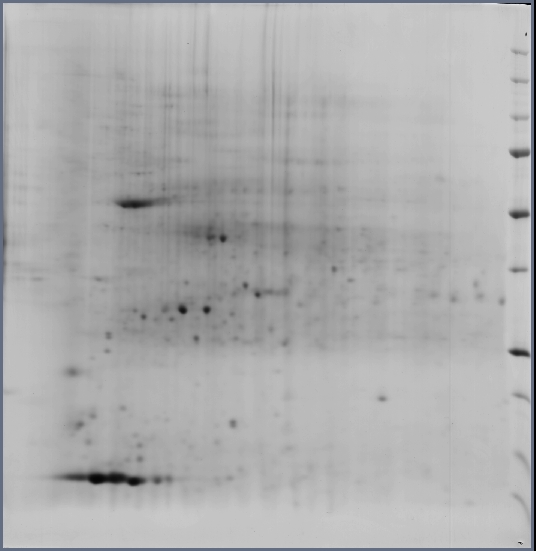


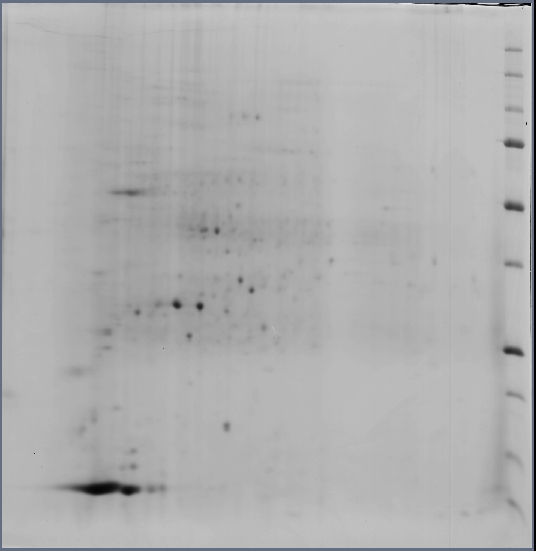


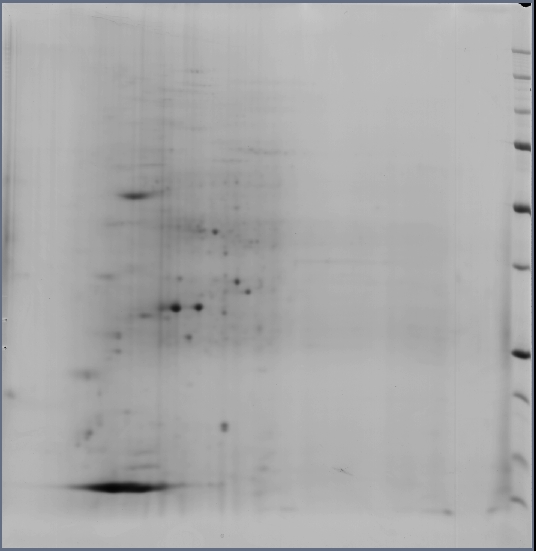


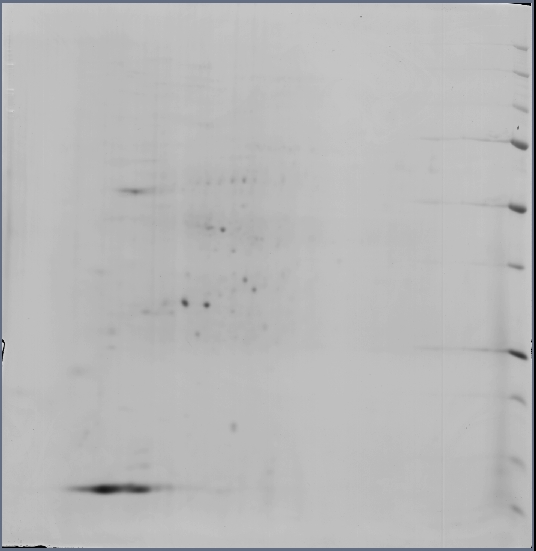

Supplement: Additional file 1: — Comparison of four gels run using the same sample from a Swedish red cow. (DOCX 404 kb) [file 12866_2016_917_MOESM1_ESM.docx]
